# Supplementary figures and images for: Participant perceptions and experiences of a novel community-based respiratory longitudinal sampling method in Liverpool, UK: A mixed methods feasibility study
Source: PLoS One. 2023 Nov 9;18(11):e0294133. doi: 10.1371/journal.pone.0294133 (PMC10635470; doi:10.1371/journal.pone.0294133)

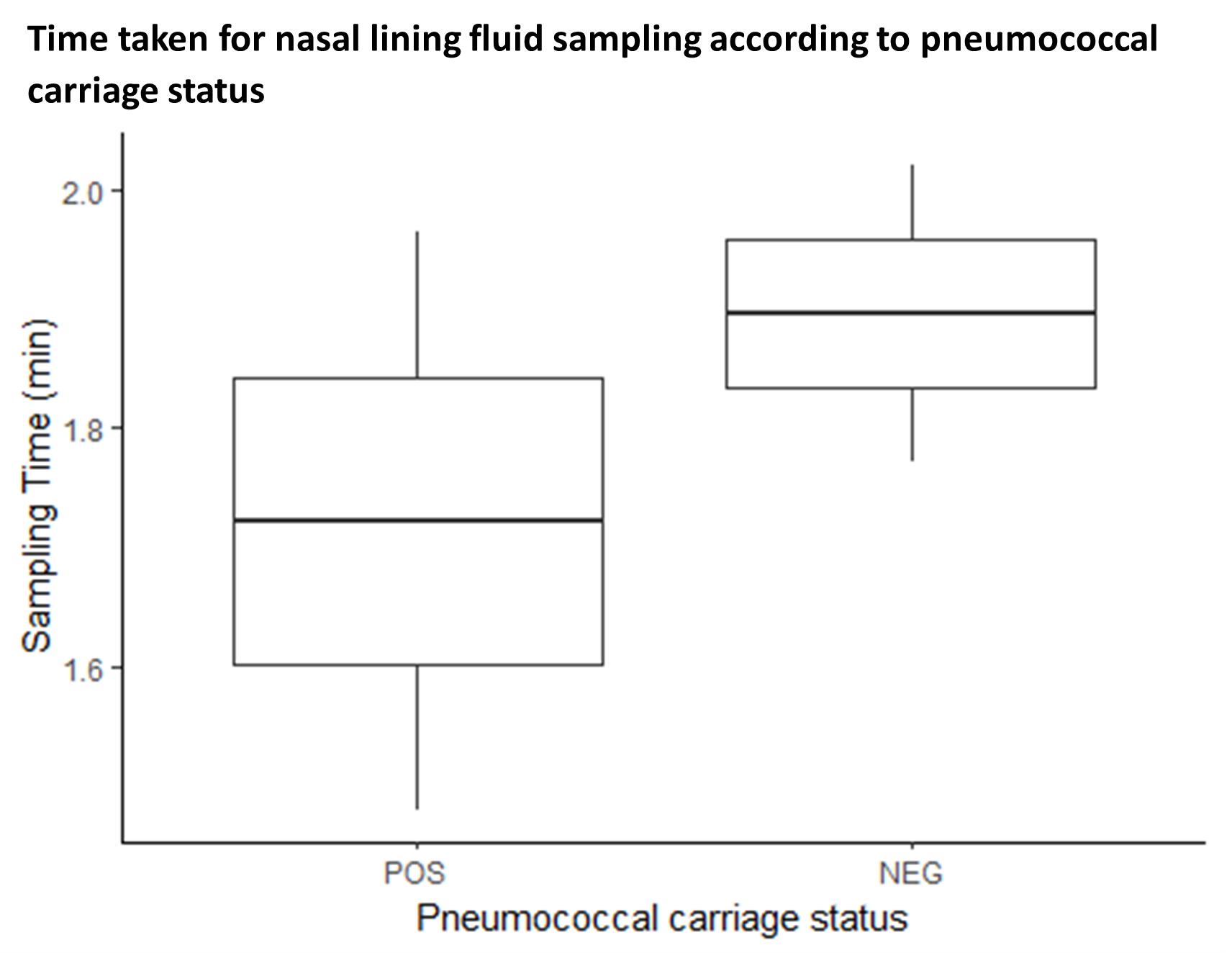

Supplement: S1 Fig — (TIF) [file pone.0294133.s003.tif]
