# Supplementary material for: Participant perceptions and experiences of a novel community-based respiratory longitudinal sampling method in Liverpool, UK: A mixed methods feasibility study
Source: PLoS One. 2023 Nov 9;18(11):e0294133. doi: 10.1371/journal.pone.0294133 (PMC10635470; doi:10.1371/journal.pone.0294133)
Supplement: S1 Checklist — (DOCX) [file pone.0294133.s005.docx]

STROBE Statement—checklist of items that should be included in reports of observational studies

|  | | | Item No. | Recommendation | Page  No. | | | Relevant text from manuscript |
| --- | --- | --- | --- | --- | --- | --- | --- | --- |
| **Title and abstract** | | | 1 | (*a*) Indicate the study’s design with a commonly used term in the title or the abstract | 1 | | | “a mixed methods feasibility study” |
|  |  |  |  | (*b*) Provide in the abstract an informative and balanced summary of what was done and what was found | 2 | | | Lines 27-45 |
| Introduction | | | | | | | |  |
| Background/rationale | | | 2 | Explain the scientific background and rationale for the investigation being reported | 2&3 | | | Lines 48-67 |
| Objectives | | | 3 | State specific objectives, including any prespecified hypotheses | 3 | | | Lines 73-75 |
| Methods | | | | | | | |  |
| Study design | | | 4 | Present key elements of study design early in the paper | 4 | | | Line 81: “a mixed methods study using a convergent parallel design”  Lines 84-85 |
| Setting | | | 5 | Describe the setting, locations, and relevant dates, including periods of recruitment, exposure, follow-up, and data collection | 4 | | | Line 90 |
| Participants | | | 6 | (*a*) *Cohort study*—Give the eligibility criteria, and the sources and methods of selection of participants. Describe methods of follow-up  *Case-control study*—Give the eligibility criteria, and the sources and methods of case ascertainment and control selection. Give the rationale for the choice of cases and controls  *Cross-sectional study*—Give the eligibility criteria, and the sources and methods of selection of participants | 4&5 | | | Line 89  Lines 102&103 |
|  |  |  |  | (*b*) *Cohort study*—For matched studies, give matching criteria and number of exposed and unexposed  *Case-control study*—For matched studies, give matching criteria and the number of controls per case |  | | | N/A |
| Variables | | | 7 | Clearly define all outcomes, exposures, predictors, potential confounders, and effect modifiers. Give diagnostic criteria, if applicable | 5 | | | Quantitative: Lines 111-116  Qualitative: Lines 127-130 |
| Data sources/ measurement | | | 8* | For each variable of interest, give sources of data and details of methods of assessment (measurement). Describe comparability of assessment methods if there is more than one group | 5&6 | | | Quantitative: Lines 111-116  Qualitative: Lines 127-135 |
| Bias | | | 9 | Describe any efforts to address potential sources of bias | 5&6 | | | Quantitative: Lines 114&115  Qualitative: Lines 133-144 |
| Study size | | | 10 | Explain how the study size was arrived at | 4&5 | | | Lines 83&84  Lines 127-129 |
| Quantitative variables | | 11 | | Explain how quantitative variables were handled in the analyses. If applicable, describe which groupings were chosen and why | 5&6 | | Lines 117-125 | |
| Statistical methods | | 12 | | (*a*) Describe all statistical methods, including those used to control for confounding | 5 | | Lines 118-121 | |
|  |  |  |  | (*b*) Describe any methods used to examine subgroups and interactions |  | | N/A | |
|  |  |  |  | (*c*) Explain how missing data were addressed | 5&6 | | Lines 123-125 | |
|  |  |  |  | (*d*) *Cohort study*—If applicable, explain how loss to follow-up was addressed  *Case-control study*—If applicable, explain how matching of cases and controls was addressed  *Cross-sectional study*—If applicable, describe analytical methods taking account of sampling strategy | 5&6 | | Lines 123-125 | |
|  |  |  |  | (*e*) Describe any sensitivity analyses |  | | N/A | |
| Results | | | | | | | | |
| Participants | | 13* | | (a) Report numbers of individuals at each stage of study—eg numbers potentially eligible, examined for eligibility, confirmed eligible, included in the study, completing follow-up, and analysed | 7&8 | | Quantitative: Lines 148-150  Qualitative: Lines 186&187 | |
|  |  |  |  | (b) Give reasons for non-participation at each stage |  | | Not known | |
|  |  |  |  | (c) Consider use of a flow diagram |  | | N/A | |
| Descriptive data | | 14* | | (a) Give characteristics of study participants (eg demographic, clinical, social) and information on exposures and potential confounders | 7&8 | | Quantitative: Lines 148-150; Table 1  Qualitative: Lines 186&187  Full study participant characteristics can be found in the related manuscript | |
|  |  |  |  | (b) Indicate number of participants with missing data for each variable of interest | 7&8 | | Quantitative: Lines 148-150; Table 1  Qualitative: Lines 186&187 | |
|  |  |  |  | (c) *Cohort study*—Summarise follow-up time (eg, average and total amount) |  | | Full details can be found in the related manuscript | |
| Outcome data | | 15* | | *Cohort study*—Report numbers of outcome events or summary measures over time | 7&8 | | Quantitative: Lines 148-150; Table 1  Qualitative: Lines 186&187 | |
|  |  |  |  | *Case-control study—*Report numbers in each exposure category, or summary measures of exposure |  | | N/A | |
|  |  |  |  | *Cross-sectional study—*Report numbers of outcome events or summary measures |  | | N/A | |
| Main results | | 16 | | (*a*) Give unadjusted estimates and, if applicable, confounder-adjusted estimates and their precision (eg, 95% confidence interval). Make clear which confounders were adjusted for and why they were included | 7&8 | | Lines 154-181 | |
|  |  |  |  | (*b*) Report category boundaries when continuous variables were categorized |  | | N/A | |
|  |  |  |  | (*c*) If relevant, consider translating estimates of relative risk into absolute risk for a meaningful time period |  | | N/A | |
| Other analyses | 17 | | Report other analyses done—eg analyses of subgroups and interactions, and sensitivity analyses | | 8-14 | Qualitative: Lines 187-321 | | |
| Discussion | | | | | | | | |
| Key results | 18 | | Summarise key results with reference to study objectives | | 14&15 | Lines 324-350 | | |
| Limitations | 19 | | Discuss limitations of the study, taking into account sources of potential bias or imprecision. Discuss both direction and magnitude of any potential bias | | 15&16 | Lines 356-371 | | |
| Interpretation | 20 | | Give a cautious overall interpretation of results considering objectives, limitations, multiplicity of analyses, results from similar studies, and other relevant evidence | | 16&17 | Lines 381-386 | | |
| Generalisability | 21 | | Discuss the generalisability (external validity) of the study results | | 16 | Lines 372-379 | | |
| Other information | | |  | | | | | |
| Funding | 22 | | Give the source of funding and the role of the funders for the present study and, if applicable, for the original study on which the present article is based | | 17 | Lines 397-400 | | |

*Give information separately for cases and controls in case-control studies and, if applicable, for exposed and unexposed groups in cohort and cross-sectional studies.
